# Root exudates and chemotactic strains mediate bacterial community assembly in the rhizosphere soil of *Casuarina equisetifolia* L

**DOI:** 10.3389/fpls.2022.988442

**Published:** 2022-09-20

**Authors:** Qi Lin, Miaomiao Li, Ying Wang, Zhixia Xu, Lei Li

**Affiliations:** Ministry of Education Key Laboratory for Ecology of Tropical Islands, Hainan Normal University, Haikou, China

**Keywords:** *casuarina equisetifolia* L., bacterial diversity, chemotaxis, root exudates, hainan

## Abstract

Rhizosphere bacterial diversity and community structure are important factors involving in plant growth. However, the exact process of how plant rhizosphere bacterial community structures is assembled remains unclear. To investigate the role of bacterial chemotaxis to rhizosphere secretions in the establishment of rhizosphere microbial community in *Casuarina equisetifolia*, we screened bacteria strains derived from the rhizosphere of *Casuarina equisetifolia* L. using top three chemicals of the plant root exudates (2,4-di-tert-butylphenol, methyl stearate, and arginine) as chemoattractant. Among 72 bacterial strains, five showed strong chemotaxis to 2,4-di-tert-butylphenol, six to methyl stearate, and eleven to arginine, with the highest bacterial chemotaxis occurring at a concentration of 60 μM. This indicates that arginine is a more important chemoattractant than 2,4-di-tert-butylphenol, methyl stearate in the establishment of rhizosphere microbial community in *Casuarina equisetifolia*. Bacterial community assembly analysis using different chemoattractants and chemoattractants-plus-bacteria combinations were then performed by burying laboratory prepared bags of sterlized soil into *C. equisetifolia* forest. Bacteria diversity and enrichment analyses using 16S rDNA sequencing at 7 and 14 days after burying showed that arginine-plus-*Ochrobactrum* sp. and *Pantoea* sp. treatment exhibited the greatest similarity to the natural forest bacterial community. Our date provides new insights into how chemoattractants and chemotactic bacteria strains shape the rhizosphere microbial community of *C. equisetifolia*, which constitutes foundational information for future management of these communities.

## Introduction

Plants secrete signaling molecules to attract beneficial microorganisms to aggregate in the rhizosphere and colonize specific plant chambers, forming a second genome; they then apply their immune systems and provide specific nutrients and habitats to promote growth or prevent soilborne diseases (Lugtenberg and Kamilova, 2009; Cordovez et al., 2019). Thus, the study of the growth and development of plants *via* plant microorganisms is increasingly viewed as a feasible approach (Geddes et al., 2015), but the prerequisite is a basic understanding of how plants regulate the assembly of rhizobacterial communities.

Some studies have shown that root exudates (REs) can mediate interactions between rhizosphere components (Vives-Peris et al., 2020), and recruit beneficial soil bacteria to shape rhizosphere microbiomes (Feng et al., 2019). Some specific compounds can also act as signaling molecules that regulate rhizosphere microbial activity (Sasse et al., 2018; Wang et al., 2019). For example, nitrogen deficiency in legumes increases in the flavonoids content of REs in order to attract rhizobia for colonization and thereby increase nitrogen availability (Badri and Vivanco, 2009). However, the mechanisms by which REs promote microbial aggregation remain largely unknown.

Bacterial chemotaxis to rhizosphere exudates may be the main mechanism driving microbial aggregation (Erhardt, 2016). Chemotaxis, the movement of organisms toward or away from chemicals, is an important adaptive behavior in motile bacteria. Bacteria use chemotaxis to participate in host colonization (Erhardt, 2016; Johnson and Ottemann, 2018), biofilm formation (He and Bauer, 2014), and quorum sensing (Rader et al., 2011) when forming symbiotic relations with plants (Scharf et al., 2016). Chemotaxis allows bacteria to obtain more favorable environmental conditions as well as better sources of carbon and nitrogen. Generally, chemicals that promote chemotaxis in bacteria are small molecules such as organic acids, fatty acids, amino acids, lipids, and secondary metabolites (Feng et al., 2019). For example, *Bacillus amyloliquefaciens* T-5 showed strong chemotaxis toward malic acid, citric acid, succinic acid, and fumaric acid, which are produced by plants to recruit bacterial biocontrol agents for pest and disease resistance (Tan et al., 2013). *Pseudomonas fluorescens* WCS365 showed strong chemotaxis toward small-molecule organic acids (such as citric acid, succinic acid, and fumaric acid) and amino acids (such as aspartic acid, glutamic acid, and isoleucine) secreted by tomato roots, which serves to promote the growth and development of the plant (de Weert et al., 2002). However, whether REs can act as chemoattractants and how they affect the assembly of plant rhizobacteria communities are poorly understood.

*Casuarina equisetifolia* L. was introduced in Guangdong Province, China, at the end of 1950 and has since become an important tree species in southeastern China (Kang and Zhong, 1999). In particular, *C. equisetifolia* provides a defense against natural disasters in coastal areas and facilitates ecological remediation, as this species supports abundant endophytes and rhizosphere microorganisms (Lin et al., 2008). However, the existence of allelopathy renders *C. equisetifolia* self-renewal difficult, reducing its potential functionality (Li et al., 2015). Previously, we analyzed the microbial diversity in the rhizosphere of *C. equisetifolia* and isolated 72 strains of culturable bacteria from rhizosphere soil and plant roots, identifying several endophytes and rhizosphere microorganisms involved in the allelopathy of *C. equisetifolia* (Huang, 2019; Zhang et al., 2020). We also found relatively high levels of 2,4-di-tert-butylphenol, methyl stearate, and arginine in *C. equisetifolia* root and litter exudates, and demonstrated that whereas 2,4-di-tert-butylphenol and methyl stearate had strong allelopathic effects (Huang, 2019; Zhang et al., 2020), arginine serves as a nutrient resource of endophytes and rhizosphere microorganisms (Huang, 2019; Zhang et al., 2020). However, it is unknown whether 2,4-di-tert-butylphenol, methyl stearate, and arginine, as allelopathic substances and nutrients, can be involved in bacterial community assembly in the rhizosphere soil of *C. equisetifolia* as chemoattractors.

To investigate the role of bacterial chemotaxis to rhizosphere secretions in the establishment of rhizosphere microbial community in *Casuarina equisetifolia*, we used 2,4-di-tert-butylphenol, methyl stearate, and arginine to screen for chemoattractant effects of rhizosphere bacteria of *C. equisetifolia* in this study, and then we tried to elucidate their functionality as chemoattractants and the factors contributing to rhizobacterial community assembly. First, different concentrations of 2,4-di-tert-butylphenol, methyl stearate, and arginine as chemoattractants were used, and by the swarming assay, we screen for chemotactic strains among the previously identified 72 culturable strains. Subsequently, to determine the effects of chemoattractants and chemotactic strains on bacterial community assembly, “chemoattractant+sterile soil” and “chemoattractant+chemotactic strains+sterile soil” models were placed in the rhizospheres of *C. equisetifolia* in the field, and evaluated the resulting communities using 16S rRNA gene profiling and bioinformatics analysis. The findings from this study will elucidate the relationship between allelochemicals and nutrients and the *C. equisetifolia* microbiome, and provide comprehensive evidence regarding how chemoattractants and chemotactic strains shape microbial communities of tree rhizosphere, which constitutes important foundational information for future management and regulation of these communities.

## Materials and methods

### Experimental materials and reagents

The field trial was established in the Haikou Guilinyang Economic Development Zone in Hainan Province (20°01′02′′N, 110°31′20′′E), China. This site has a tropical marine monsoon climate with mean annual precipitation of 1,500–2,000 mm. The sampling site has a mean annual temperature of up to 23.8°C and a long sunshine duration (Cai et al., 2010). A total of 72 strains of culturable bacteria were isolated from the rhizosphere of *C. equisetifolia* (**Table S1**). All chemicals (>98% purity) were purchased from Sangon Biotech (Shanghai, China).

### Measurement of bacterial chemotaxis with different simulated exudates

Bacterial chemotaxis was measured *via* a swarming assay modified from that described by (Englert et al., 2009), using semi-solid 0.5% agar culture medium. Luria–Bertani (LB) semi-solid culture medium containing different concentrations (0, 30, 60, and 90 μM) of 2,4-di-tert-butylphenol, methyl stearate, or arginine was sterilized (121°C, 20 min), poured into sterile, 90 mm diameter petri dishes, and dried in a laminar flow cabinet for 20 min. Sterile filter paper (8 mm diameter) was placed in the center of each plate. Concomitantly, 72 strains of bacteria that were activated overnight were subcultured to sterile LB culture medium (without agar) at 37°C with shaking at 180 rpm until reaching an OD_600_ of about 0.8. Bacterial suspension (2 μl) was added to the sterile filter paper, then plates were cultured at 28°C for 2 d. Diameters (cm) of bacterial colonies were measured in different directions, bacteria with a chemotaxis ring mean diameter >2 cm were defined as a strongly chemotactic strain (He et al., 2017). The assay was replicated thrice.

### Experimental design to determine the effects of different simulated exudates and chemotactic strains on the assembly of forest bacterial communities

**Figure 1:**
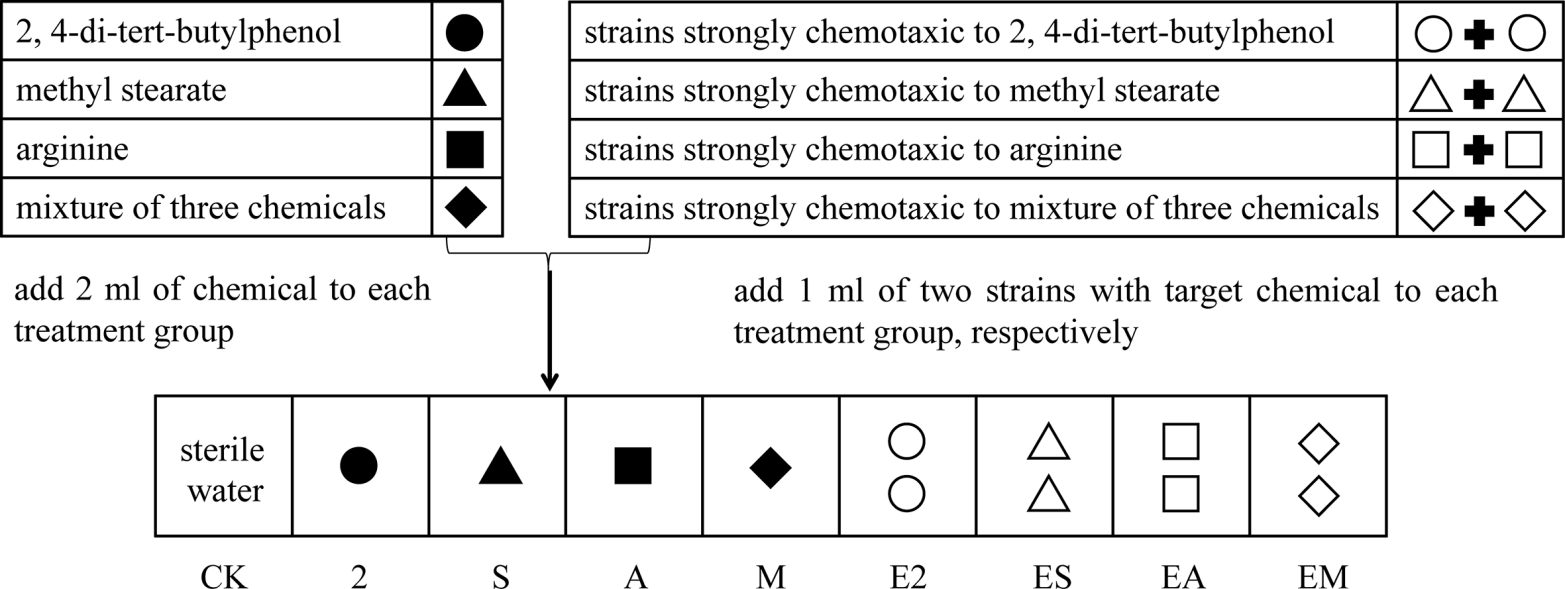
Experimental design to determine the effects of different simulated chemoattractants and chemotactic strains on bacterial community assembly in the rhizosphere soils of a *C. equisetifolia* forest.

As the substrate for bacterial community assembly, 50 g *Casuarina equisetifolia* forest soil was added to nonwoven fabric bags (dimension: 8 cm × 10 cm) and sterilized (121°C, 60 min). Then, 2 ml of each sterilized chemotaxis agent, 2, 4-di-tert-butylphenol, methyl stearate, or arginine, or a mixture of the three agents was added to the bagged sterile soil (treatments 2, S, A, and M, respectively). In addition, suspensions (OD_600_ of 0.8) of two bacteria identified as strongly chemotactic to each chemotaxis agent were centrifuged (600 × *g*, 3 min), the supernatants discarded, and the obtained pellets resuspended in 1 ml of the sterile target chemical and added to the bagged sterile soil. The eight strongly chemotactic strains were mixed with chemoattractant agents respectively: *Enterobacter hormaechei* strain AMS-38 and *Acinetobacter nosocomialis* strain AC1530, strongly chemotactic to 2,4-di-tert-butylphenol with (treatments E2), *Enterobacter* sp. and *Enterobacter cloacae*, strongly chemotactic to methyl stearate (treatments ES), *Ochrobactrum* sp. and *Pantoea* sp., strongly chemotactic to arginine (treatments EA), and *Bacillus cereus* strain CP1 and *Pseudomonas* sp., strongly chemotactic to a mixture of the three chemoattractants (treatments EM). The final concentration of the chemoattractant in the all treatments was 60 μM. An equivalent amount of sterile (2 ml) distilled water was used as the blank control (CK). The bags were buried in rhizosphere soil (depth of approximately 25 cm) surrounding six *C. equisetifolia* trees randomly selected in October, 2021. Half of the bags were removed after 7 d, and the other half after 14 d. Simultaneously, *C. equisetifolia* rhizosphere soil was collected as another control group (R). The detailed experimental design is shown in **Figure 1**. Each treatment had three replicates.

### Sequencing to determine bacterial diversity

A FastDNA^®^ Spin Kit for Soil (MP Biomedicals LLC, Santa Ana, CA, USA) was used to extract the total DNA of soil bacterial genomes from the samples collected in Section 2.3. Primers 338F (5′-ACT CCT ACG GGA GGC AGC AG-3′) and 806R (5′-GGA CTA CHV GGG TWT CTA AT-3′) were used for PCR amplification of the bacterial 16S rRNA gene. The PCR reaction mixture contained 12.5 μl of Premix Taq DNA polymerase (Takara, Dalian, China), 0.5 μl (200 μM) of each primer, and 10 ng of template DNA, with PCR-grade water added to a final volume of 25 µl. The PCR amplification cycling conditions were as follow: initial denaturation at 94°C for 2 min; 30 cycles of denaturing at 94°C for 30 s, annealing at 55°C for 30 s, and extension at 72°C for 45 s; and a final 10 min elongation at 72°C. Sequencing was performed on an Illumina MiSeq PE300 platform (Majorbio, Shanghai, China).

### Bioinformatics analyses

The 300 bp reads were truncated at any site receiving an average quality score below 20; truncated reads shorter than 50 bp were discarded, as were reads containing ambiguous characters. The resultant sequences were assembled according to their overlapped sequence with a minimum of 10bp; the maximum mismatch ratio of an overlapping region was 0.2, and reads that could not be assembled were discarded. Then, the optimized sequences were clustered into operational taxonomic units (OTUs) using UPARSE 7.1 with 97% sequence similarity level (Edgar, 2013). The most abundant sequence for each OTU was selected as a representative sequence. The taxonomy of each OTU representative sequence was analyzed using RDP Classifier (v.2.2) against the 16S rRNA gene database (Silva v.138) (Wang et al., 2007). Nonbacterial OTUs (e.g., mitochondrial, chloroplast, and viridiplantae) were removed.

### Statistical analyses

Analysis of variance (ANOVA) was conducted on the data collected in Section 2.2 using SPSS v19 software (IBM, Armonk, NY, USA). Results are expressed as the mean ± standard error (SE). Mothur (v.1.30.2, the University of Michigan, Ann Arbor, MI, USA) was used for alpha index analysis of the data collected in Section 2.3, and a nonparametric statistical test (Kruskal–Wallis test) was used to evaluate the alpha-diversity difference. Networkx (v.1.11, the University of California, Davis, CA, USA) was used to construct species correlation networks by calculating Spearman correlations between species. R (v.3.3.1, https://cran.r-project.org/src/base/R-3/R-3.3.1.tar.gz) was used to examine similarity or difference in community composition by computing weighted UniFrac distance matrices; data were ordinated using non-metric multidimensional scaling (NMDS), Venn diagram analysis of numbers of OTUs, and community histogram analysis based on data tables in the tax_summary_a folder. The stats package in R (v.3.3.1) and the scipy package in Python (v.2.7, Amsterdam, The Netherlands) were used for the analysis of significant differences in species between different treatments using the Kruskal–Wallis test.

## Results

### Chemotaxis of bacteria under the action of specific components in the solution

**Figure 2:**
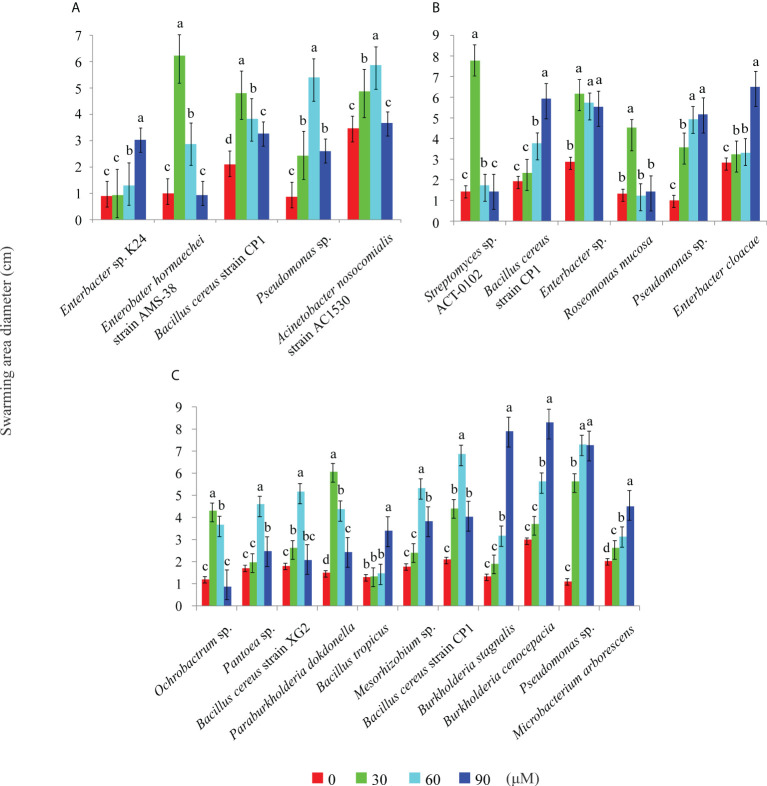
Effect of chemoattractants on the swarming motility of different bacteria. Swarming diameters were measured for chemoattractants at concentrations of 0, 30, 60, and 90 μM. **(A)**: 2, 4-di-tert-butylphenol; **(B)**: methyl stearate; **(C)**: arginine. Different letters above the columns indicate significant difference (*P < *0.05).

**Figure 3:**
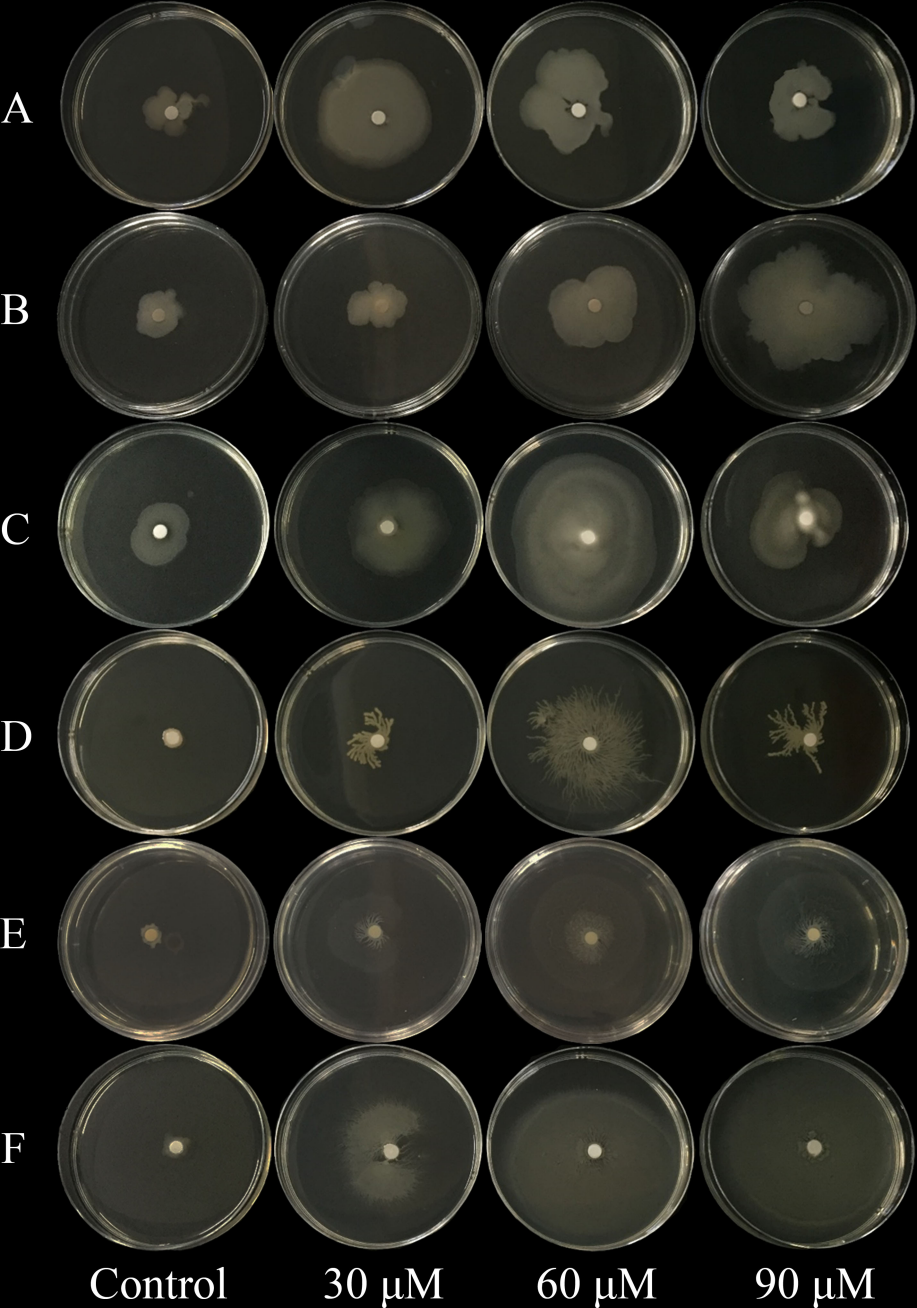
Effect of chemoattractants on the swarming motility of *Bacillus cereus* strain CP1 and *Pseudomonas* sp. **(A–C)** *Bacillus cereus* strain CP1; **(D–F)**: *Pseudomonas* sp. **(A, D)** Chemoattractant 2, 4-di-tert-butylphenol; **(B, E)** chemoattractant methyl stearate; **(C, F)** chemoattractant arginine. Chemoattractant concentration from left to right: 0, 30, 60, and 90 μM.

The effects of 2,4-di-tert-butylphenol, methyl stearate, and arginine, selected as representative RE chemoattractants of *C. equisetifolia*, on chemotaxis of bacteria strains were examined. Among 72 bacterial strains tested, five showed strong chemotaxis to 2,4-di-tert-butylphenol such as *Enterobacter hormaechei* strain AMS-38, etc (**Figure 2A**), six to methyl stearate such as *Enterobacter* sp., etc (**Figure 2B**), and 11 to arginine such as *Ochrobactrum* sp., etc (**Figure 2C**). The highest number of bacterial strains was observed to show chemotaxis at chemoattractant concentrations of 60 μM. This indicates that arginine is a more important chemoattractant than 2,4-di-tert-butylphenol, methyl stearate in the establishment of rhizosphere microbial community in *C. equisetifolia*. *Bacillus cereus* strain CP1 and *Pseudomonas* sp. showed strong chemotaxis to all of the chemoattractants (**Figures 3A**–**F**).

### Effects of different chemoattractants and chemotactic strains on bacterial community assembly

Based on the results from Section 3.1 (**Figures 2**, **3**), the following strains were selected to determine the effects of different simulated exudates and chemotactic strains on the assembly of forest bacterial communities: *Enterobacter hormaechei* strain AMS-38 and *Acinetobacter nosocomialis* strain AC1530, with strong chemotaxis to only 2,4-di-tert-butylphenol (E2); *Enterobacter* sp. and *Enterobacter cloacae*, with strong chemotaxis to only methyl stearate (ES); *Ochrobactrum* sp. and *Pantoea* sp., with strong chemotaxis to only arginine (EA); and *Bacillus cereus* strain CP1 and *Pseudomonas* sp., with strong chemotaxis to all three compounds (EM).

**Figure 4:**
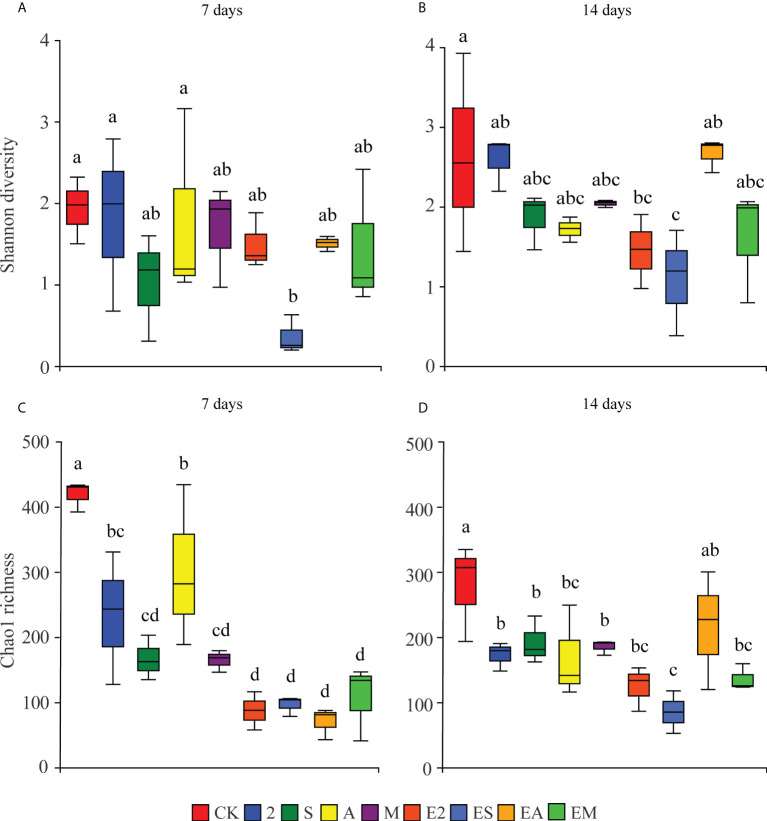
Box plots of the effects of chemoattractants and chemoattractants-plus-chemotactic strains on the Chao1 richness and Shannon diversity of bacterial genera on days 7 **(A, C)** and 14 **(B, D)**. Different lowercase letters indicate significant differences among treatments (*P < *0.05).

Alpha diversity analysis following 16S rRNA gene profiling of the bacterial community in each group revealed lower richness and diversity of bacteria in the experimental treatment groups than those in CK following both 7 and 14 days of treatment (**Figure 4**). Larger differences were observed in bacterial richness and diversity among chemoattractant treatment groups on day 7 than on day 14. In contrast, the differences among chemoattractant+chemotactic strain treatment groups were greatest on day 14 (**Figures 4A–D**). Moreover, compared with other treatments, both at 7 and 14 days, bacterial richness and diversity in S and ES treatments were lower. This may mean that methyl stearate and its chemotactic bacteria play a weak role in the recruitment of microorganisms.

**Figure 5:**
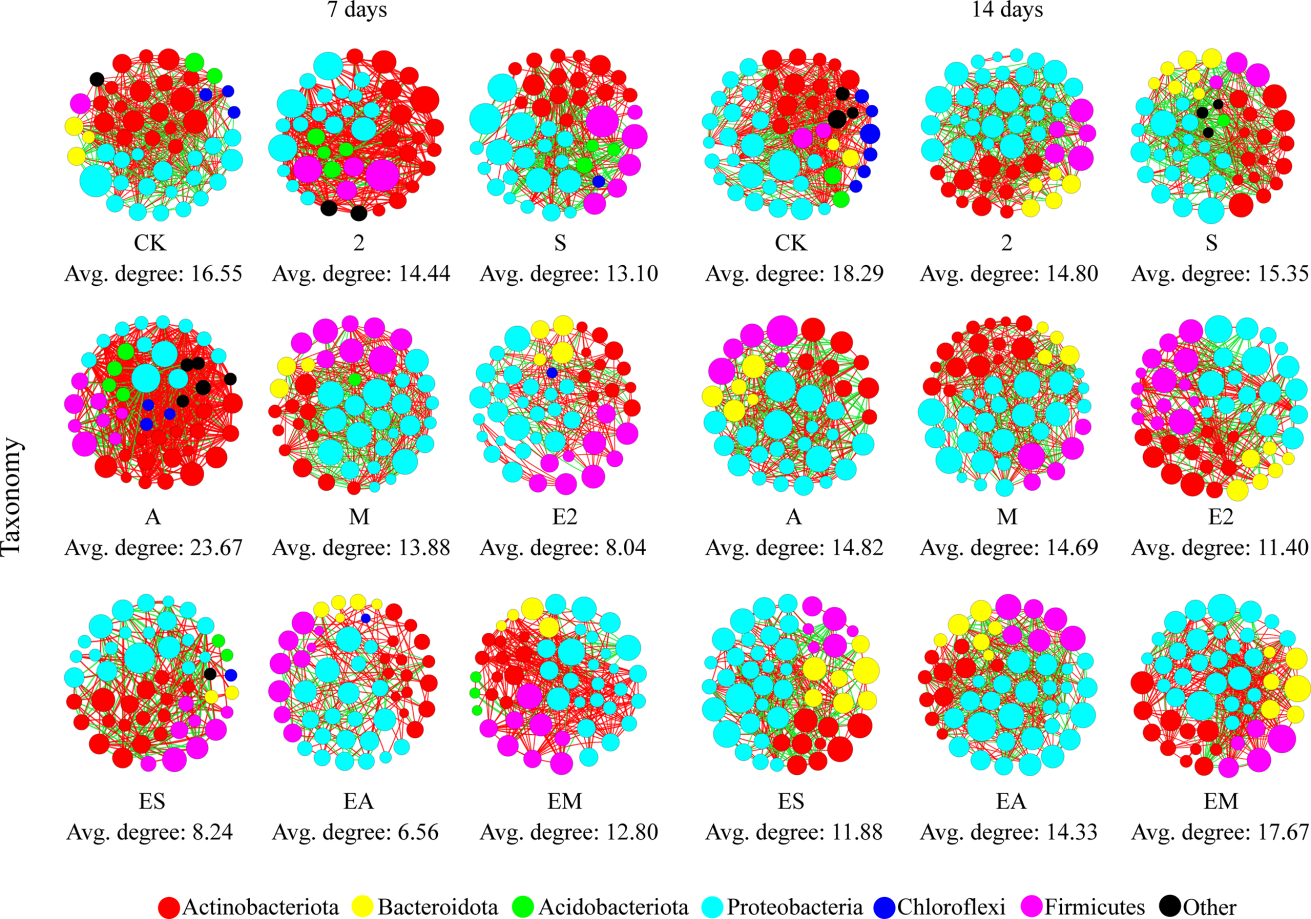
Bacterial co-occurrence networks as affected by chemoattractant and chemoattractant-plus-chemotactic strain treatments at 7 and 14 days at the phylum level. Avg, Average degree of taxonomy.

Average degree is used to indicate the degree of network complexity among the bacteria in the treatment. On day 7, the network complexity of all treatment groups, except A, decreased compared with that in CK (**Figure 5**). The lowest network complexity occurred following EA treatment (Avg. degree: 6.56). On day 14, the network complexity of all treatment groups decreased compared with that of CK. The lowest network complexity was found in the E2 group (Avg. degree: 11.40). Larger increases over time were detected following treatments including chemotactic strains compared with those following only chemoattractant treatment. There results suggest that chemoattractant mainly act in the prophase (7 days), and chemotactic bacteria mainly act in the later phase (14 days) in bacterial community.

### Effects of different chemoattractants and chemotactic strains on bacterial community recruitment

**Figure 6:**
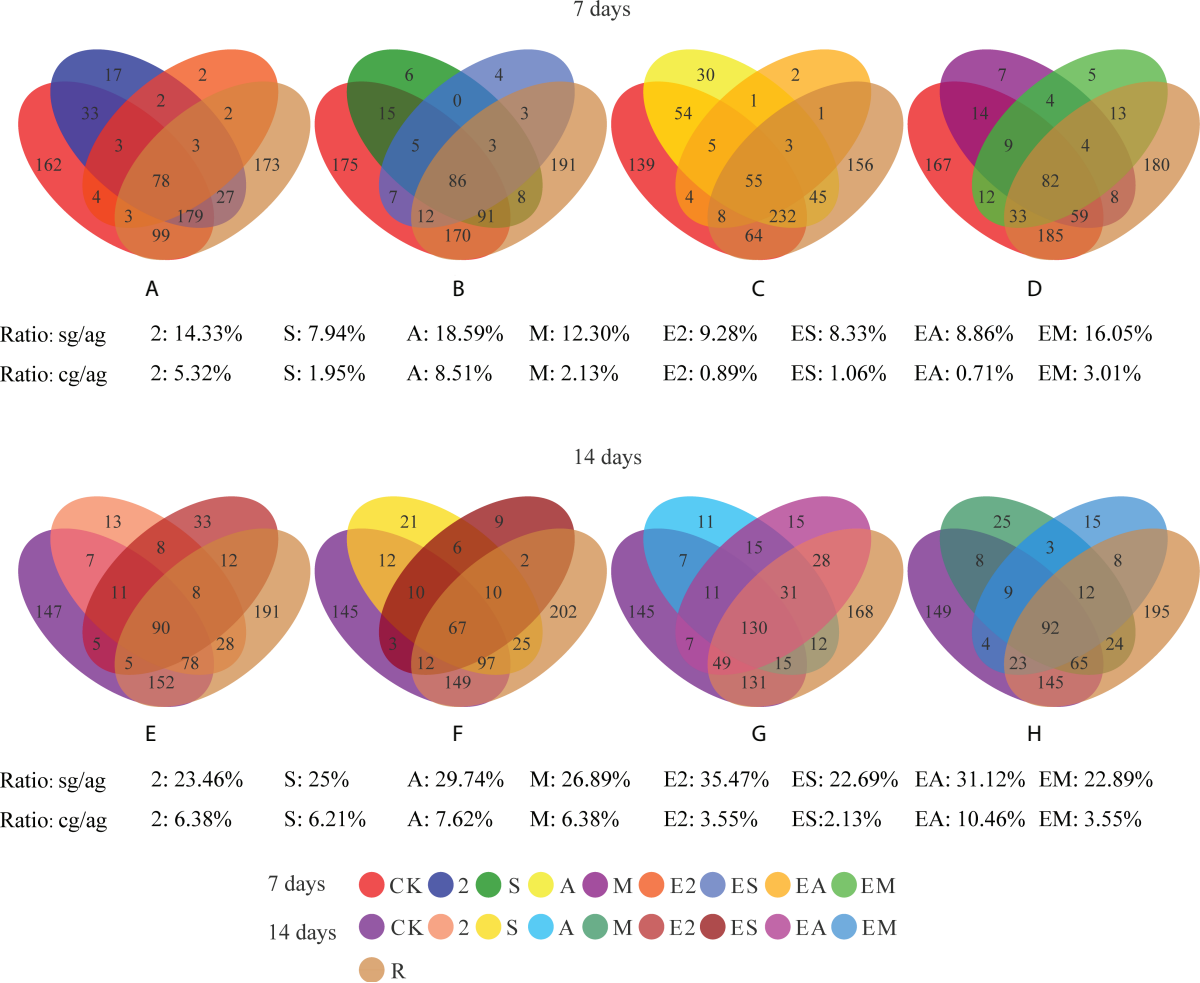
Venn diagrams showing the numbers of shared and unique bacterial genera in chemoattractant and chemoattractant-plus-chemotactic strain treatments at 7 **(A–D)** and 14 **(E–H)** days and in rhizosphere soils of a *C. equisetifolia* forest. sg, specific genera. cg, common genera. ag, all genera.

Venn diagrams were used to reveal the differences in bacterial species between the chemoattractant and the chemoattractant+chemotactic strain treatment groups (**Figures 6A–H**). From the number of specific genera in each treatment group shown in the Venn, we calculated the ratio of the number of specific genera (compared with CK) in each treatment group to the number of all genera in the treatment group (hereinafter referred to as “Ratio: sg/ag”) to reflect the ability of different treatment groups to recruit bacteria.

In comparisons of bacterial genera between chemoattractant treatment groups and CK on day 7 (**Figures 6A–D**), the proportion of specific bacteria species was highest (ratio sg/ag: 18.59%) in treatment group A, and lowest (ratio sg/ag: 7.94%) in group S. On day 14 (**Figures 6E–H**), the proportion of specific bacteria species in group A remained highest (ratio sg/ag: 29.74%), although that in group S increased considerably (ratio sg/ag: from 7.94 to 25%).

In comparisons of bacterial genera between chemoattractant+chemotactic strain treatment groups and CK on day 7 (**Figures 6A–D**), the proportion of specific bacteria species was highest (ratio sg/ag: 16.05%) in EM and lowest (ratio sg/ag: 8.33%) in ES. On day 14 (**Figures 6E–H**), the proportion was highest in the E2 group (ratio sg/ag: 35.47%).

In addition, recruitment capacity of the different treatments increased with time. Chemoattractant treatments exhibited greater recruitment capacity than chemoattractant+chemotactic strain treatments on day 7, whereas on day 14, chemoattractant+chemotactic strain treatments showed greater recruitment capacity. These results are consistent with the results in **Figures 4**, **5**, which together show that chemotactic bacteria, rather than chemoattractants, play a key role in the later stages (14 days).

**Figure 7:**
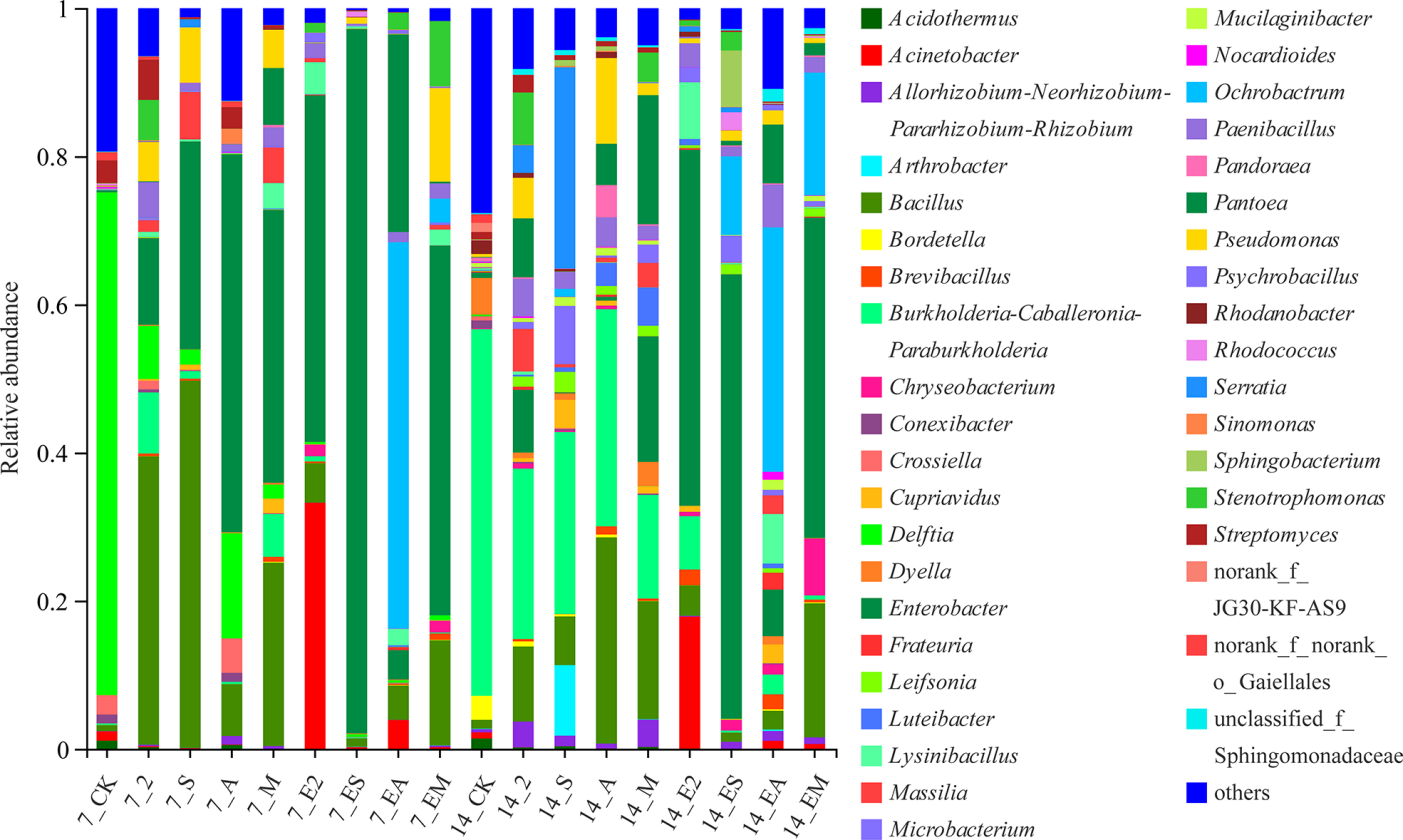
Relative abundance of bacterial genera following chemoattractant and chemoattractant-plus-chemotactic strain treatments at 7 and 14 days.

**Figure 7** illustrates the differences in the relative abundance of bacteria between the various treatment groups. On day 7, the relative abundances of *Enterobacter* (11.70, 28.06, 51.02, and 36.84%) and *Bacillus* (38.93, 49.59, 7.01, and 24.76%) were increased in chemoattractant treatment groups 2, S, A, and M, respectively, compared with those in CK; however, the relative abundances of these two strains decreased on day 14 in all four treatment groups. In contrast, the relative abundance of *Burkholderia–Caballeronia–Paraburkholderia* increased in the four groups with time (day 7: 8.24, 1.03, 0.30, and 5.79%; day 14: 23.01, 24.58, 29.30, and 13.97%, respectively).

**Figure 8:**
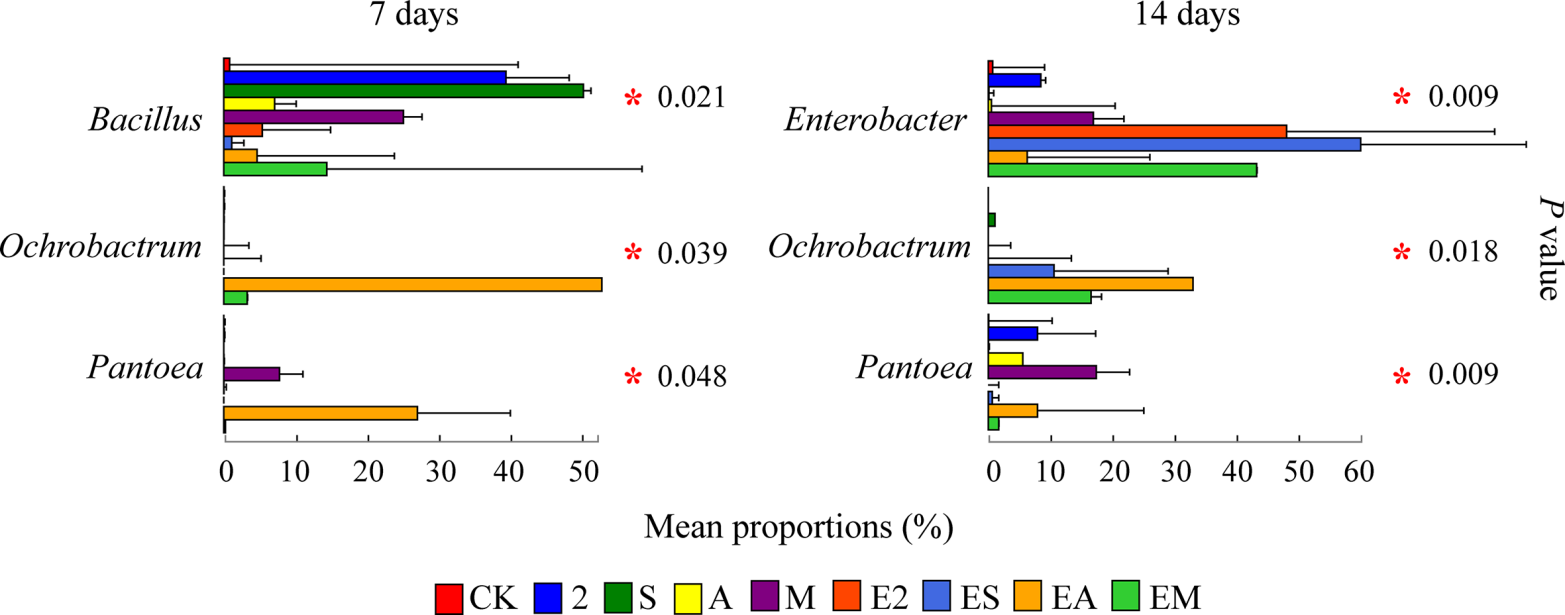
Proportions of bacterial genera with high relative abundances in chemoattractant and chemoattractant-plus-chemotactic strain treatment groups at 7 and 14 days. Asterisks in red indicate a significant difference among treatments (*P < *0.05).

After the difference analysis, we found that some genera with high abundance different in treatment groups on days 7 and 14, and between chemoattractant treatment groups and chemoattractant+chemotactic treatment groups (**Figure 8**). On day 7, the relative abundances of *Bacillus*, *Ochrobactrum*, and *Pantoea* were significantly different between various treatment groups. *Bacillus* showed chemotaxis to all three types of chemoattractants, its relative abundance was highest in group S, but decreased with ES. The relative abundance of *Ochrobactrum* increased in group EM than M. Relative abundance of *Pantoea* increased in group M compared with CK, but decreased in group EM. On day 14, the relative abundances of *Enterobacter*, *Ochrobactrum*, and *Pantoea* were significantly different among various treatment groups. *Enterobacter* relative abundance was increased in groups EA than A, and EM than M. The relative abundance of *Ochrobactrum* was increased in groups ES than S, and EM than M. Among chemoattractant groups, the relative abundance of *Pantoea* was enhanced in group M compared with that in groups 2, S and A. Alternatively, the relative abundance of *Pantoea* was decreased in groups EM than M.

### Similarity of bacterial communities shaped by chemoattractants and chemotactic bacteria to rhizobacterial bacterial diversity

We calculated the ratio of the number of common genera (compared with R) in each treatment group to the number of all genera in the treatment group (hereinafter referred to as “Ratio: cg/ag”) to reflect the similarity of the bacterial community in rhizosphere soil. Comparison of the common and all genera in treatment groups (**Figure 6**) with *C. equisetifolia* forest bacterial diversity (treatment R) revealed that the bacterial communities recruited by treatment A were most similar to that in treatment R on both days 7 and 14. Chemoattractant treatment groups showed an overall increase with time, with the greatest increase in group S (ratio cg/ag: from 1.95 to 6.21%). Among chemoattractant+chemotactic strain treatments, the bacterial communities recruited by EM were the most similar to those of R (ratio cg/ag: 3.01%) on day 7, whereas the EA group exhibited the most similarity to R on day 14 (ratio cg/ag: 10.46%); EA also exhibited the greatest increase in similarity (ratio cg/ag: from 0.71), although other treatment groups also showed increases over time. These results further indicate that bacterial communities shaped by arginine-plus-*Ochrobactrum* sp. and *Pantoea* sp., not only arginine, contribute more to the shaping of native bacterial communities compared with other treatment groups.

**Figure 9:**
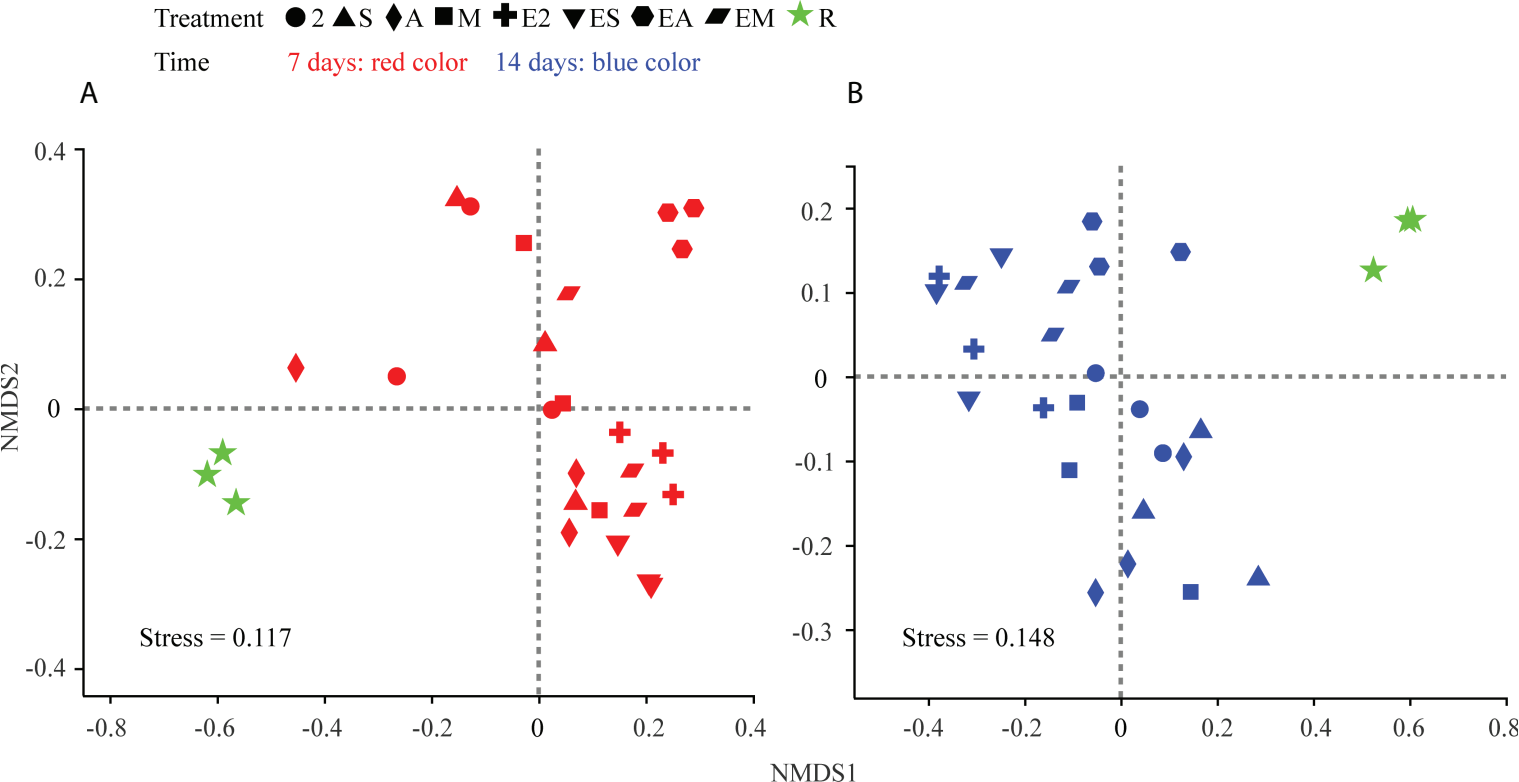
NMDS ordinations based on weighted UniFrac distance matrices of bacterial communities in chemoattractant and chemoattractant-plus-chemotactic strain treatments at 7 **(A)** and 14 **(B)** days and in rhizosphere soils of a *C. equisetifolia* forest.

The results of NMDS analysis (**Figure 9**) revealed differences among all groups at both 7 and 14 days. Chemoattractant treatment groups on the two sample dates were generally tightly clustered, as were chemoattractant+chemotactic strain treatment groups. The *C. equisetifolia* forest microflora was more similar to those following chemoattractant treatments than chemoattractant+chemotactic strain treatments; however, the microflora shaped by EA treatment was more similar to woodland bacteria on day 14 than on day 7, and also most closely resembled the forest microflora of *C. equisetifolia* (treatment R) with the passage of time, consistent with the results from genera ratio comparisons.

## Discussion

Substantial progress has been made with regard to investigating the mechanisms by which plant microorganisms promote plant growth and inhibit disease; however, the effects of plant REs on shaping rhizosphere microbial communities are poorly understood. In this study, we applied motility experiments to confirm that 2,4-di-tert-butylphenol, methyl stearate, and arginine were chemoattractants, and could be used to screen bacterial strains with strong chemotaxis to these agents individually and in combination. Our results further indicated that these chemoattractant compounds and chemotactic strains played a driving role in the bacterial community assembly of the *C. equisetifolia* rhizosphere. These results provide comprehensive, empirically based theoretical guidance for the management and manipulation of plant microbial communities in the future.

### Selection of chemoattractants and screening of chemotactic strains

REs are important in regulating rhizosphere microbial growth, chemotaxis, biofilm formation, and rhizosphere colonization (Li et al., 2017; Feng et al., 2019). Studies on chemoattractants have typically focused on small-molecule organic acids and amino acids with a few reports related to phenols and fatty acids (Li et al., 2017). In earlier work, we showed that 2,4-di-tert-butylphenol and methyl stearate could act as allelochemicals affecting the growth of *C. equisetifolia* trees and microorganisms, and we also found a high level of arginine in *C. equisetifolia* roots (Li et al., 2016; Chen, 2021). 2, 4-di-tert-butylphenol was also shown to function as a bacteriostatic agent and a chemoattractant to attract beneficial strains to prevent soil-borne diseases (Marchese et al., 2017). The present study was the first to identify 2,4-di-tert-butylphenol and methyl stearate as chemoattractants that can regulate rhizosphere microflora assembly (**Figure 2**). Previously, Moreover, although methyl stearate has not been previously reported to induce bacterial chemotaxis, palmitic acid, palmitoleic acid, stearic acid, and oleic acid in peanut REs were found to be beneficial to bacterial motility, chemotaxis, and adsorption and colonization (Cesari et al., 2019), and palmitic acid, palmitoleic acid, and stearic acid exhibit chemotactic effects on KLBMP 4941 (Xiong et al., 2020). Thus, we speculated that methyl stearate, as the product of stearic acid esterification, might also function as a chemoattractant. Together, these results indicate that phenols and fatty acids can also act as signaling molecules and participate in plant–rhizobacteria interactions.

Potentially key bacterial taxa have important ecological roles in microbiome assembly and ecosystem function (Banerjee et al., 2018). Among the chemotactic strains, we found that *Bacillus* and *Pseudomonas* exhibited strong chemotactic phenomena (**Figure 7**). The attraction is not surprising because *Bacillus* is ubiquitous in plant tissues and rhizosphere soil and is also important for biological control and bio-fertilization (Tahir et al., 2019). In addition, the application of *Bacillus* can provide protection against abiotic stress in plants and during rhizosphere microbial interactions (Feng et al., 2018). Some bacilli (such as *B. cereus*) are also antagonistic to soil-transmitted diseases (Fira et al., 2018). Our previous studies have shown that *Bacillus* is present in the non-rhizosphere soils of *C. equisetifolia*, rhizosphere soils, and roots, with high abundance in the latter (Lin, unpublished). In addition, *Bacillus* can infect *C. equisetifolia* roots (Chen, 2021). Therefore, we hypothesized that this bacterial genus underwent horizontal transmission and aggregated within the rhizosphere through chemotaxis, and then infected and proliferated in large numbers within the roots, subsequently protecting *C. equisetifolia* from soil pathogens. In comparison, *Pseudomonas* can grow in different habitats (Weller, 2007) and is usually regarded as a plant pathogen; however, it can also promote plant growth and inhibit other soil pathogens (Burr et al., 1978). Little is known regarding whether *Pseudomonas* exhibits a chemotactic response to plant secretions. In the present study, we identified that *Pseudomonas* exhibited chemotaxis toward 2, 4-di-tert-butylphenol, methyl stearate, and arginine, representative of *C. equisetifolia* REs (**Figure 7**). This suggests that *Pseudomonas* might accumulate in the rhizosphere through chemotaxis, consequently inhibiting the transmission of soil-borne pathogens and promoting plant growth. Collectively, these results indicate that plants may recruit beneficial bacteria *via* REs to enhance their overall viability.

### Effects of chemoattractants and chemotactic strains on recruitment to bacterial communities

REs and rhizobacteria are important for the assembly of rhizosphere microbiomes (Bais et al., 2006; Haggag and Timmusk, 2008). In the present study, we investigated the factors affecting rhizobacterial community assembly. Among the three chemoattractants evaluated, the arginine-regulated bacterial communities were most similar to the *C. equisetifolia* rhizosphere microbiome (**Figures 6**, **9**). Arginine exerts chemotactic effects on *Paenibacillus polymyxa* SQR21 (Shen, 2016), and, as a carbon source, is crucial for the survival of bacteria. Therefore, we speculated that microorganisms in *C. equisetifolia* forests might initially be recruited and aggregated by arginine, subsequently achieving symbiosis *via* plant–bacteria interactions. In turn, the compounds 2,4-di-tert-butylphenol and methyl stearate function both as allelochemicals and chemoattractants that can recruit bacteria to aggregate within plant rhizospheres, and jointly inhibit the growth of other plants (or species) and decrease competition for essential resources. However, few studies have evaluated allelochemicals that are also chemoattractants. Nevertheless, these results collectively indicate that REs can regulate root microbiome composition and activity in different ways that allow plants and microorganisms to form a “holobiont” symbiotic relationship (Vandenkoornhuyse et al., 2015). The phenomenon is highly relevant toward understanding how plants shape rhizosphere microbial communities.

In the present study, we found that *Enterobacter* exhibited high relative abundance in the chemoattractant treatment groups (**Figure 7**), and also showed strong chemotaxis toward chemoattractants in swarming assays (**Figure 2**). We therefore speculated that this genus might be chemotaxis by REs, and synergistically shaping the rhizosphere microbiome together with other bacteria. We also found that the bacterial communities driven by arginine-plus-*Ochrobactrum* sp. and *Pantoea* sp. were most similar to those in the rhizosphere (**Figures 6**, **9**). Although arginine might be recruited as a carbon source, few studies are available regarding *Ochrobactrum* and *Pantoea* functionality in this context. In preliminary work, we found that *Ochrobactrum* sp. and *Pantoea* sp. harbor quorum sensing related genes (Li, unpublished). Therefore, we hypothesized that various bacteria were recruited *via* specific REs through chemotaxis, which then attracted surrounding bacteria to migrate to the rhizosphere through quorum sensing signal molecules, thereby completing the bacterial community assembly process of a *Casuarina* forest. These results suggest that both chemotaxis and chemotactic bacteria play an indispensable role in shaping bacterial communities.

## Conclusions

In this study, the relative contributions of chemoattractants and chemotactic strains to the assembly of rhizobacterial communities were examined through comprehensive analysis. The compounds 2,4-di-tert-butylphenol, methyl stearate, and arginine, as representative REs, promoted bacterial motility in swarming assays, with the highest numbers of strains demonstrating strong chemotaxis when the concentration of chemoattractants was 60 μM. Arginine-plus-*Ochrobactrum* sp. and -*Pantoea* sp. treatment yielded the bacterial community most similar to that of a *C. equisetifolia* forest. The results of this study increase our understanding of the mechanisms regulating rhizosphere microbiome assembly. In addition, the results have important implications for the use of plant rhizosphere microbiomes and provide baseline data for managing non-crop rhizospheres. Future studies should focus on the changes in transcription and metabolism in microbial communities following regulation by chemoattractants in order to provide new theoretical data regarding the assembly of non-crop rhizosphere microbiomes.

## Data availability statement

The data presented in this study can be found in the NCBI SRA repository, accession number SRP396822.

## Author contributions

LL conceived the idea and designed the experiments. QL performed the experiments, analyzed the data, and wrote the paper. YW, ML, and ZX performed the experiments. All authors contributed to the article and approved the submitted version.

## Funding

This work was supported by the Innovation Platform for Academicians of Hainan Province (YSPTZX202129) and the Hainan Provincial Natural Science Foundation of China (320QN254).

## Acknowledgments

We thank Prof. Rodolfo Dirzo from the Department of Biology, Stanford University for his kind advice on data processing and manuscript writing.

## Conflict of interest

The authors declare that the research was conducted in the absence of any commercial or financial relationships that could be construed as a potential conflict of interest.

## Publisher’s note

All claims expressed in this article are solely those of the authors and do not necessarily represent those of their affiliated organizations, or those of the publisher, the editors and the reviewers. Any product that may be evaluated in this article, or claim that may be made by its manufacturer, is not guaranteed or endorsed by the publisher.
